# Analysis of gut microbiota and metabolites in patients with rheumatoid arthritis and identification of potential biomarkers

**DOI:** 10.18632/aging.203641

**Published:** 2021-10-20

**Authors:** Yumei Chen, Chiyu Ma, Lixiong Liu, Jingquan He, Chengxin Zhu, Fengping Zheng, Weier Dai, Xiaoping Hong, Dongzhou Liu, Donge Tang, Yong Dai

**Affiliations:** 1 Clinical Medical Research Center, Guangdong Provincial Engineering Research Center of Autoimmune Disease Precision Medicine, Shenzhen Engineering Research Center of Autoimmune Disease, The Second Clinical Medical College of Jinan University, The First Affiliated Hospital Southern University of Science and Technology, Shenzhen People’s Hospital, Shenzhen 518020, Guangdong, People’s Republic of China; 2 Department of Rheumatology and Immunology, The Second Clinical Medical College of Jinan University, The First Affiliated Hospital Southern University of Science and Technology, Shenzhen People’s Hospital, Shenzhen 518020, Guangdong, People’s Republic of China; 3 College of Natural Science, University of Texas at Austin, Austin, TX 78721, USA; 4 Guangxi Key Laboratory of Metabolic Disease Research, Guilin No. 924 Hospital, Guilin 541002, Nanning, People’s Republic of China

**Keywords:** rheumatoid arthritis, gut microbiota, metabolites, biomarkers

## Abstract

Rheumatoid arthritis (RA) is an autoimmune disease described by joint destruction, synovitis and pannus formation. The gut microbiota acts as an environmental factor that plays an important role in RA, but little research regarding the etiopathogenic mechanisms of the microbiome in RA has been carried out. We used an integrated approach of 16S rRNA gene sequencing and ultrahigh-performance liquid chromatography-mass spectrometry-based metabolomics to analyze the structure and diversity of the intestinal flora and metabolites of the gut microbiota in RA patients compared with healthy subjects. In this study, α-diversity analysis of the gut microbiota showed that there was no significant difference between the healthy control (HC) and RA groups. However, β-diversity analysis showed that there was a significant difference between the two groups. Further analysis of alteration of the gut microbiota revealed that at the phylum level, the relative abundance of p_Bacteroidetes was significantly decreased in the RA group, while that of Verrucomicrobia and Proteobacteria was significantly increased in the RA group. At the genus level, Bacteroides, Faecalibacterium and some probiotics were decreased in the RA group, while 97 genera, including *Lactobacillus*, *Streptococcus* and *Akkermansia*, were increased in the RA group. Seventy-four differentially abundant metabolites were identified between the HC and RA groups, and we identified two potential biomarkers (9,12-octadecadiynoic acid and 10Z-nonadecenoic acid) in RA.

## INTRODUCTION

Rheumatoid arthritis (RA) is a systemic autoimmune disease characterized by chronic inflammation of the joints, bone erosion and cartilage destruction [1]. In 2015, RA was reported to affect approximately 24.5 million people worldwide [2]. The incidence of RA in China is approximately 0.37%, and the total number of RA patients is approximately 5 million [3]. Although major progress has been made in the treatment and prognosis of RA, the relief rate is only 8.6%. The cost of drug treatment and the economic loss caused by loss of the labor force are tremendous burdens on the families of patients with RA and society.

Increasing evidence has shown that an imbalance in the gut microbiota leads to the occurrence and development of RA and other rheumatic diseases [4, 5]. Autoimmune disease may proceed through a pathogenic mechanism in which the gut microbiota and their metabolites regulate immune function [6, 7]. At present, whether rheumatoid arthritis is related to an imbalance in the gut microbiota remains controversial [8, 9]. However, many bacteria, including Bacteroides [10]; pathogens, such as *Prevotella copri* [11]; and probiotics, such as Bifidobacterium [12], are thought to be related with the progress of RA. Several mechanisms leading to immune abnormalities in RA have been reported; these mechanisms include the production of citrullinated peptides by *Porphyromonas gingivalis* [13] and the activation of immune responses via Th17 cells by *P. copri* [14]. In mice, host intestinal Th17 lymphocytes are induced by commensal bacteria and segmented filamentous bacteria (SFB). The introduction of SFB into germ-free mice caused the induction of lamina propria Th17 cells, the production of autoantibodies, and arthritis [15]. However, the etiology of RA linked to the microbiome remains unknown.

To analyze the structure and diversity of the intestinal flora in patients with RA and identify different metabolites in feces, we extracted total DNA from the fresh feces of 30 healthy controls and 29 patients with RA for 16S rRNA gene sequencing and metabolic spectrum analysis via ultra-high-performance liquid chromatography-mass spectrometry (UHPLC-MS). We found some differences in the intestinal flora. In addition to candidate biomarkers that may be strongly associated to the incidence and development of RA, differentially abundant metabolites may be valuable for the early diagnosis and treatment of RA.

## RESULTS

### Clinical information

The RA patients and healthy subjects were South Chinese individuals with similar eating habits to exclude dietary differences. The clinicopathological variables of the HC and RA groups were generally matched. The ages and triglyceride, total cholesterol, creatinine, and serum albumin levels in the HC and RA groups are shown in Table 1.

**Table 1:** The clinical index of healthy controls (HC) and RA patients (RA).

**Clinical index**	**HC (*n*=30)**	**RA (*n*=29)**
Age (Years)	44.1±1.9070	58.9±2.5930
Triglyceride (mol/L)	1.505±0.2373	1.289±0.1599
Total cholesterol (mol/L)	4.555±0.1494	4.429±0.1777
Creatinine (μmol/L)	75.43±3.2420	61.36±2.7270
Serum albumin (g/L)	44.76±0.5605	34.24±0.9881
HDL cholesterol (mol/L)	1.467±0.0597	1.199±0.0822
LDL cholesterol (mol/L)	2.472±0.1149	2.659±0.1306
Globulin (g/L)	26.56±0.7955	34.38±1.4650
Urea nitrogen (mmol/L)	4.78±0.2252	5.378±0.4498
Uric acid (μmol/L)	339.5±15.6500	297.1±14.8400
Total bilirubin	14.54±1.1490	7.117±0.6790

Values are mean ± standard deviation; HDL, high density lipoprotein; LDL, low density lipoprotein.

### Sequencing depth and diversity analysis of the gut microbiota

**Figure 1:**
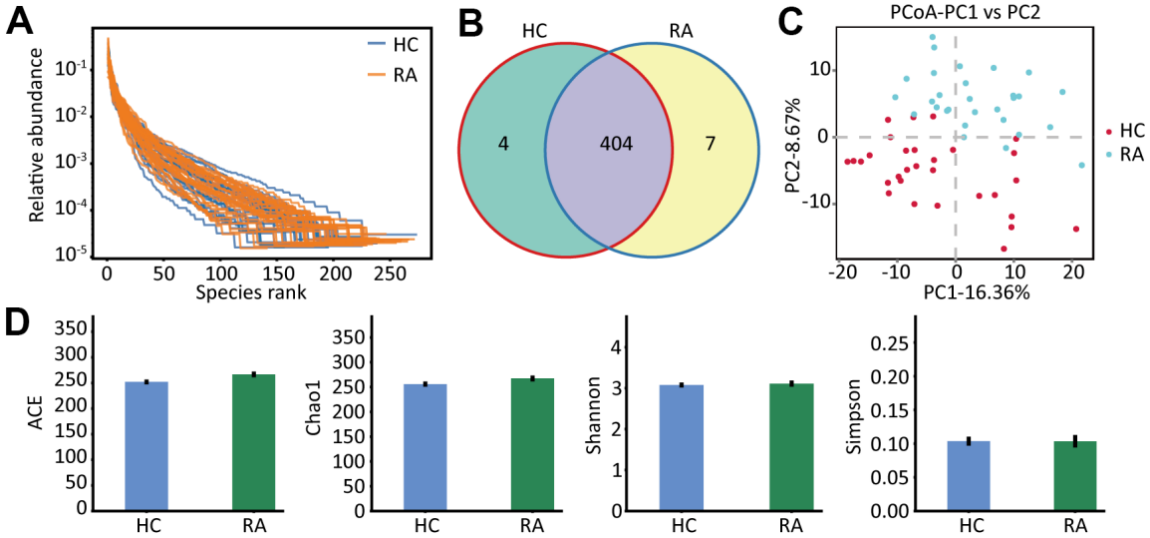
**The species abundance and diversity.** (**A**) The rank abundance curve of HC and RA group. (**B**) The OTUs (97% similarity) of HC and RA group. (**C**) The principal coordinate analysis score (PcoA) plots base on the relative abundance of OTUs, unweighted unifrac PcoA plots between HC group and RA group. HC group showed in red dots, RA group showed in blue dots. (**D**) Species α-diversity differences between the HC group and RA group (ACE, Chao1, Shannon and Simpson index).

Gut microbiota profiles of the healthy subjects and the RA patients were analyzed by 16S rRNA gene sequencing. We observed that both the richness and the evenness of the gut microbiota were high in the HC and RA groups (Figure 1A), indicating that we can obtain sufficient sequencing information from these samples in the two groups. After data filtering, we obtained 4241288 clean tags, 2177148 of which were from the HC group (range, 68085 to 74519) and 2064140 of which were from the RA group (range, 50304 to 76306). Then, we carried out operational taxonomic units (OUTs) taxonomic analysis and acquired 408 and 411 OTUs from the HC group and RA group, respectively, and among these OTUs, 404 OTUs were shared by the HC and RA groups (Figure 1B). Principal coordinate analysis (PCoA) score analysis showed that the HC and RA samples were distributed in two groups (Figure 1C). The α-diversity analysis of the ACE, Chao1, Shannon and Simpson indices showed no significant differences between the HC and RA groups (Figure 1D). Specifically, Chao1 and Ace reflect the species richness of samples, namely, the amount of species. Shannon and Simpson indices reflect the species diversity of the samples. However, β-diversity analysis showed significant differences between the HC and RA groups, which was significant according to PERMANOVA (p = 0.001) and ANOSIM analysis (p =0.001), as showed in Supplementary Figure 1.

### Composition analysis of the gut microbiota

**Figure 2:**
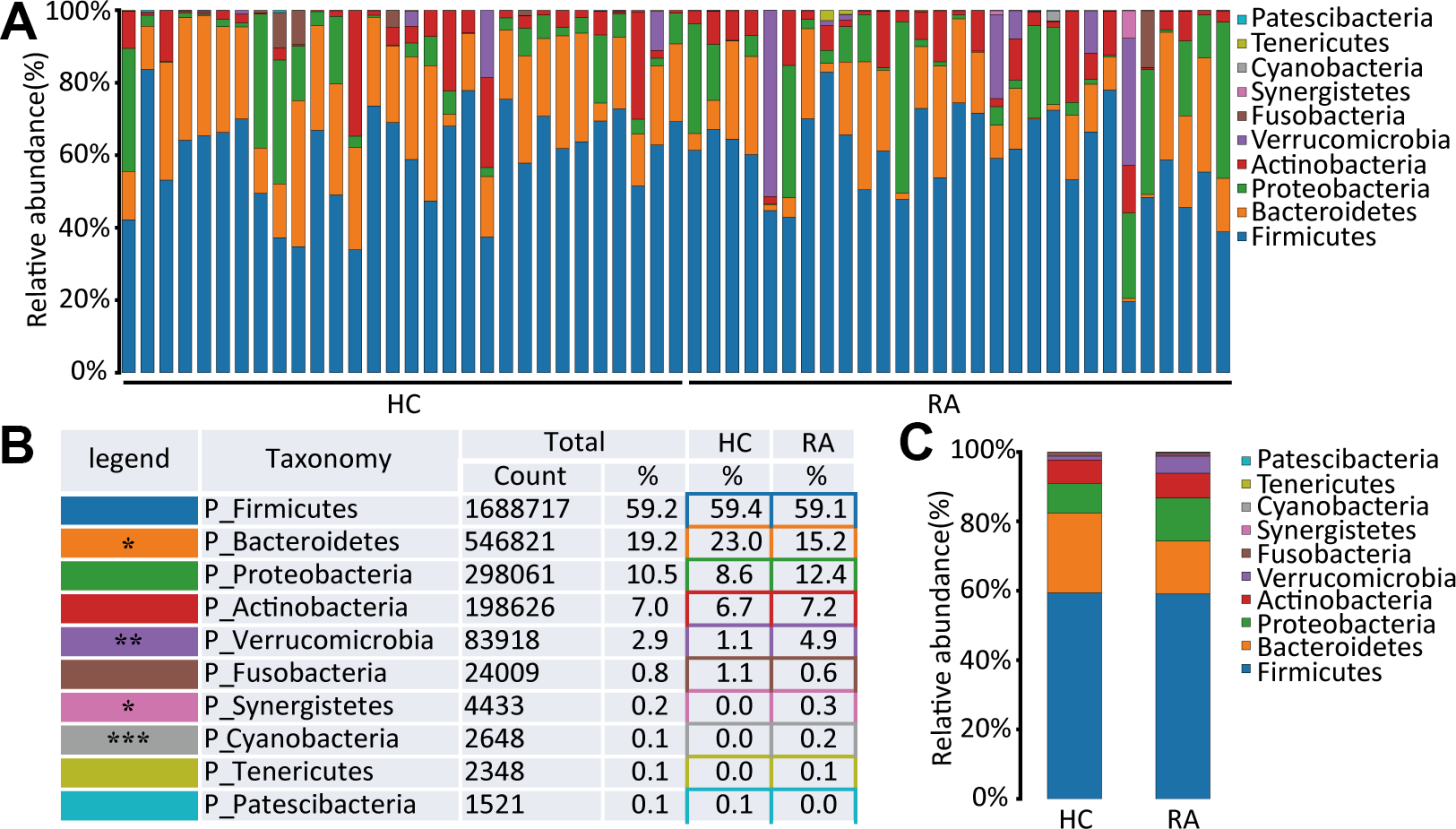
**The species abundance at phylum level.** (**A**) Relative abundance of gut microbiota in every samples at phylum level, n=30 for HC group and n=29 for RA group. (**B**, **C**) Component proportion of gut microbiota at phylum level in HC and RA group.

A total of 10 phyla in the intestinal flora were identified by 16S rRNA sequencing of the HC and RA groups. Firmicutes, Bacteroidetes, Proteobacteria and Actinobacteria were the main components of the HC and RA groups, as shown in Figure 2A. Firmicutes was the most predominant phylum, accounting for 59.4% and 59.1% of the species identified in the HC and RA groups, respectively. Bacteroidetes was more abundant in the HC group than in the RA group (23.0% versus 15.2%, respectively, p=0.0132), while Proteobacteria (8.6% versus 12.4% in the HC and RA groups, respectively) and Verrucomicrobia (1.1% versus 4.9% in the HC and RA groups, respectively, p=0.0075) were less abundant in the HC group than in the RA group (Figure 2B, 2C). Then, to assess differences in the microbiota of the HC and RA groups, we conducted a Wilcoxon test. According to the criterion of a p value<0.05, we found that Bacteroidetes, Cyanobacteria, Synergistetes and Verrucomicrobia were significantly different phyla between the HC and RA groups.

**Figure 3:**
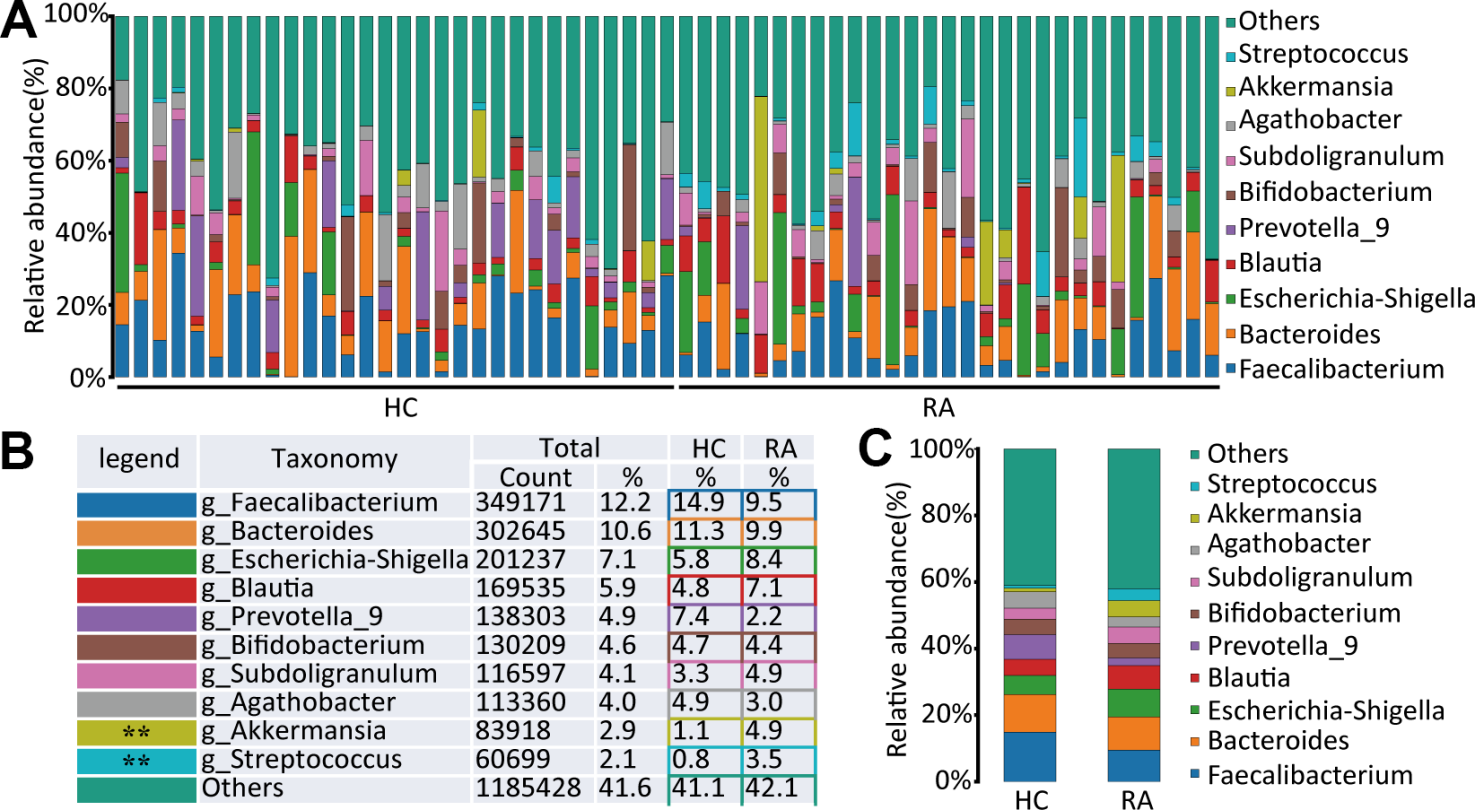
**The species abundance at genus level.** (**A**) Relative abundance of gut microbiota in every samples at the genus level, n=30 for HC group and n=29 for RA group. (**B**, **C**) Component proportion of gut microbiota at the genus level in HC and RA group.

At the genus level, Faecalibacterium, Bacteroides, Escherichia-Shigella, Blautia, Prevotella_9, Bifidobacterium, Subdoligranulum, Agathobacter, Akkermansia, and Streptococcus were the main components of the HC and RA groups, as shown in Figure 3A–3C. Of them, Streptococcus (0.8% versus 3.5%, respectively, p=0.0013) and Akkermansia (1.1% versus 4.9%, respectively, p=0.0093) were significantly different genera between the HC and RA groups. A total of 159 bacterial genera were obtained between the HC and RA groups, of which 62 genera were less abundant in the RA group, including g_uncultured_bacterium_f_Prevotellaceae, g_Prevotella, g_Faecalibacterium, g_Bifidobacterium, g_Bacteroides, g_Ruminococcus_1, g_Ruminococcaceae_UCG-002, g_Lachnospiraceae_UCG-001, and g_Fusobacterium, while 97 genera were more abundant in the RA group, including g_Lactobacillus, g_Streptococcus, g_Akkermansia, g_Blautia, and g_Eggerthella. In addition, 43 of 159 genera were significantly different (p<0.05) between the HC and RA groups (Supplementary Table 1).

### Screening the differentially abundant gut microbiota caused by RA disease

**Figure 4:**
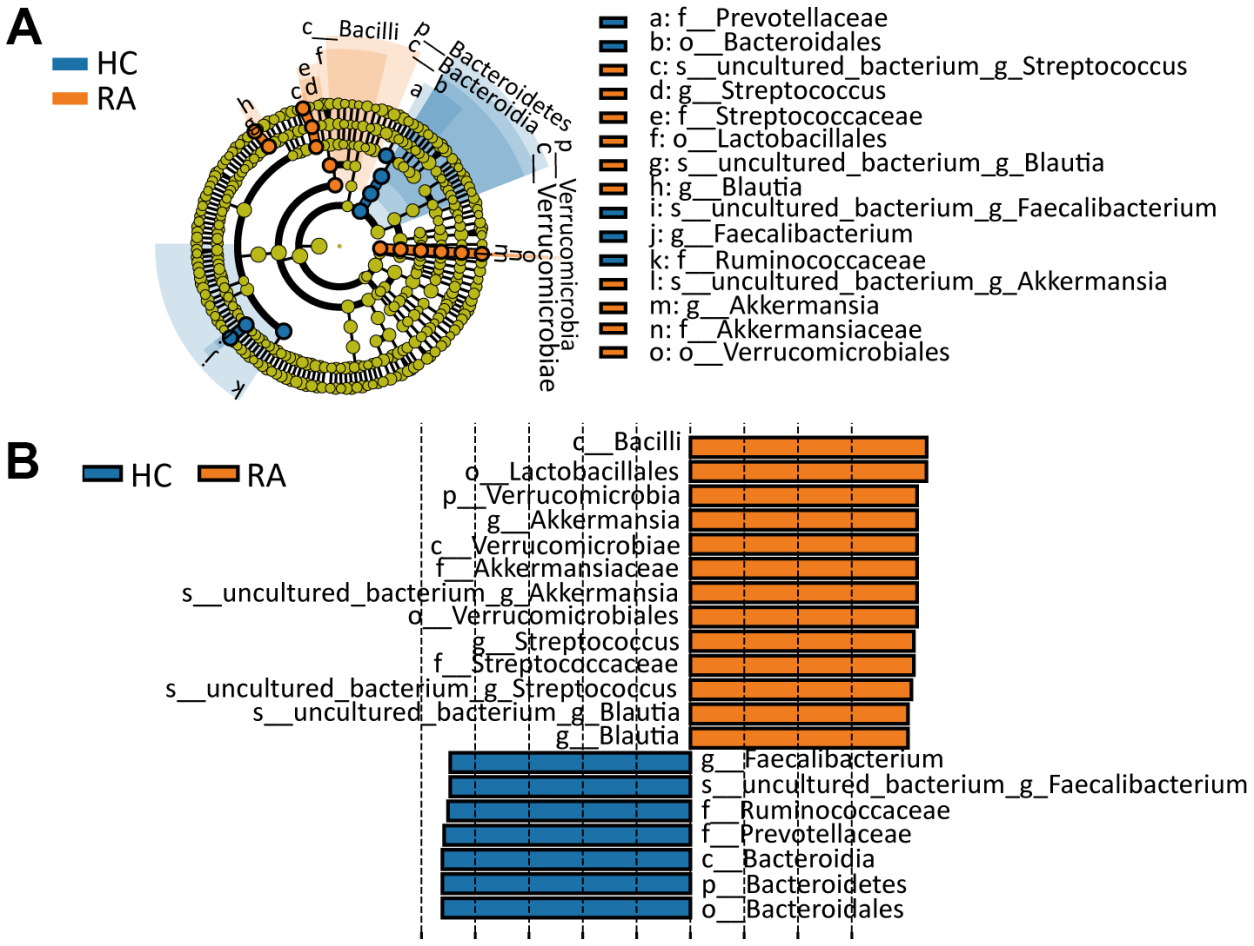
**Line discriminant analysis (LDA) effect size analysis.** (**A**) Cladogram indicating the phylogenetic distribution of differential gut microbiota between the HC or RA group. (**B**) The differential gut microbiota with LDA Score >4 between the HC and RA groups.

By the LEfSe (linear discriminant analysis effect size) method [16] and according to the filter criterion of “LDA score>4”, biomarkers with significant differences between the HC and RA groups were identified. In the cladogram and histogram in Figure 4A, 4B, circles moving from the inside to the outside of the cladogram represent the taxonomic level from phylum to species, and the diameter of each small circle is proportional to the relative abundance in the gut microbiota. The letters p, c, o, f, g and s represent phylum, class, order, family, genus and species, respectively. From the phylum to species classification levels, a total of 20 significantly different components of the intestinal flora (LDA score>4) between the HC and RA groups were identified. Among these components, p_Verrucomicrobia, c_Verrucomicrobiae, c_Bacilli, o_Verrucomicrobiales, o_Lactobacillales, f_Streptococcaceae, f_Akkermansiaceae, g_Blautia, g_Akkermansia, g_Streptococcus, s_uncultured_bacterium_g_Blautia, s_uncultured_bacterium_g_Akkermansia and s_uncultured_bacterium_g_Streptococcus were more abundant in the RA group, while p_Bacteroidetes, c_Bacteroidia, o_Bacteroidales, f_Ruminococcaceae, f_Prevotellaceae, g_Faecalibacterium and s_uncultured_bacterium_g_Faecalibacterium were more abundant in the HC group.

### Multivariate analysis of fecal metabolite profiles

Fecal metabolic profiles of the HC and RA groups were obtained by UHPLC-MS. In the Orthogonal projections to latent structures discriminant analysis (OPLS-DA) model, the fecal samples were clearly separated, with a difference in the metabolic profiles of the HC group and RA group found in both negative ion mode (NEG) and positive ion mode (POS), as shown in Supplementary Figure 2A, 2B. This finding suggests that rheumatoid arthritis can lead to significant changes in metabolites. Then, we screened out differential metabolites between the HC and RA groups and displayed them by volcano plot, as showed in Supplementary Figure 1C, 1D. And red dot indicated that compare with HC group, metabolites were up-regulated in RA group, blue dot indicated that metabolites were down-regulated in RA group.

### Identification of differentially abundant metabolites between the HC and RA groups

**Figure 5:**
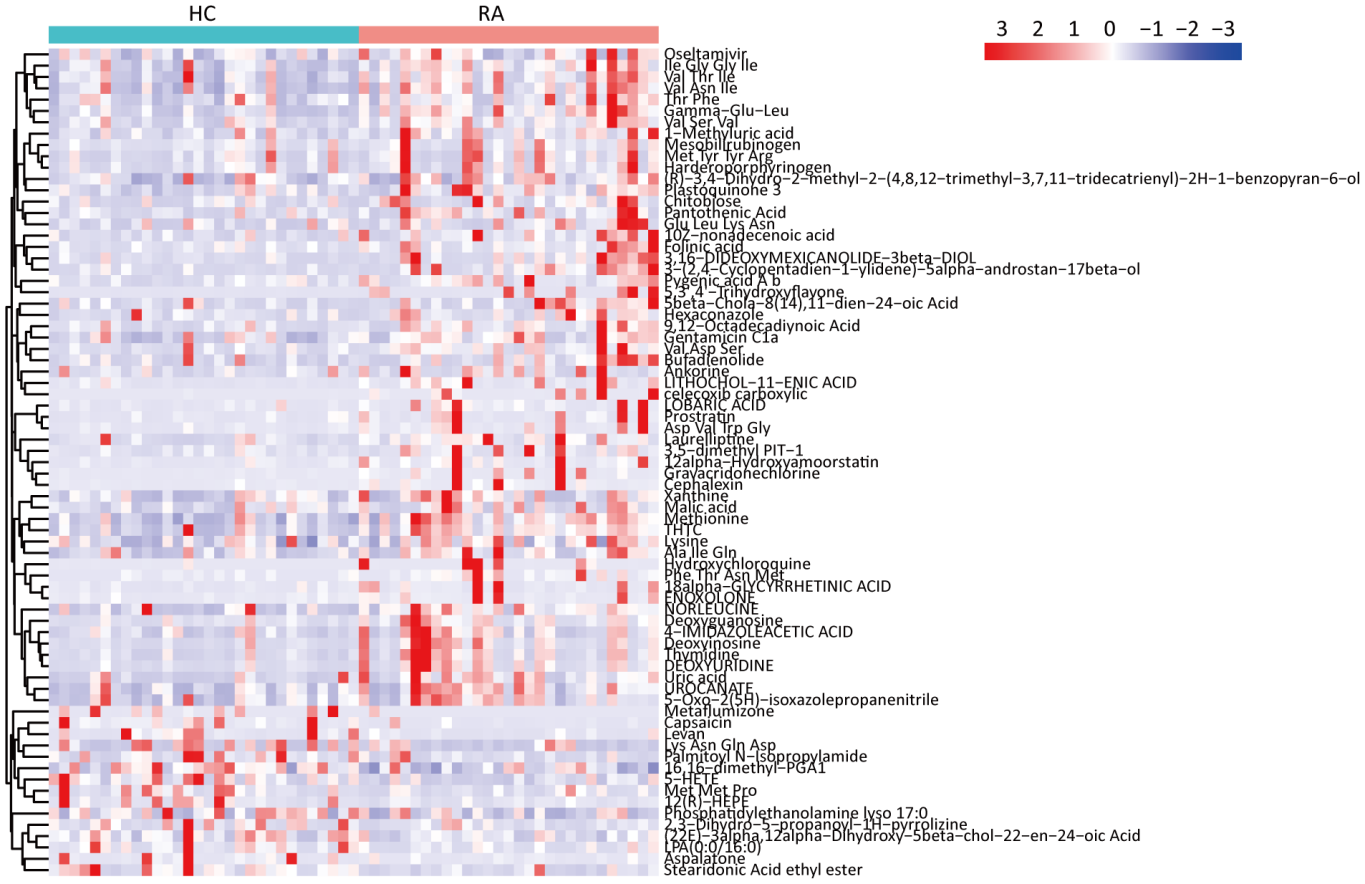
Significantly changed metabolites heatmap in feces samples between HC and RA group.

The 16S rRNA gene sequencing results showed that some components of the fecal microbiota significantly differed between the HC and RA groups; thus, we inferred that alterations in the fecal microbiota may lead to alterations in fecal metabolites. Then, we performed UHPLC-MS analyses and successfully obtained 279 metabolites in the NEG mode and 1045 metabolites in the POS mode (Supplementary Table 1). According to the conditions “VIP > 1 and P < 0.05”, 29 differentially abundant metabolites between the HC and RA groups were acquired in negative ion mode; 3 of these metabolites were decreased, while 26 metabolites were increased. In positive ion mode, 45 differentially abundant metabolites between the HC and RA groups were acquired; 12 of these metabolites were decreased, while 33 metabolites were increased. We discovered that some peptides (Val Thr Ile, Ile Gly Gly Ile, Val Asn Ile, etc.), amino acids (lysine, methionine) and nucleotides (thymidine, deoxyuridine, deoxyinosine, deoxyguanosine, etc.) were increased in the RA group compared with the HC group; however, some lipids (LPA(0:0/16:0), 16,16-dimethyl-PGA, 1,5-HETE, 12(R)-HEPE, etc.) were decreased in the RA group compared with the HC group (Figure 5).

### Pathways analysis of differentially abundant metabolites

**Figure 6:**
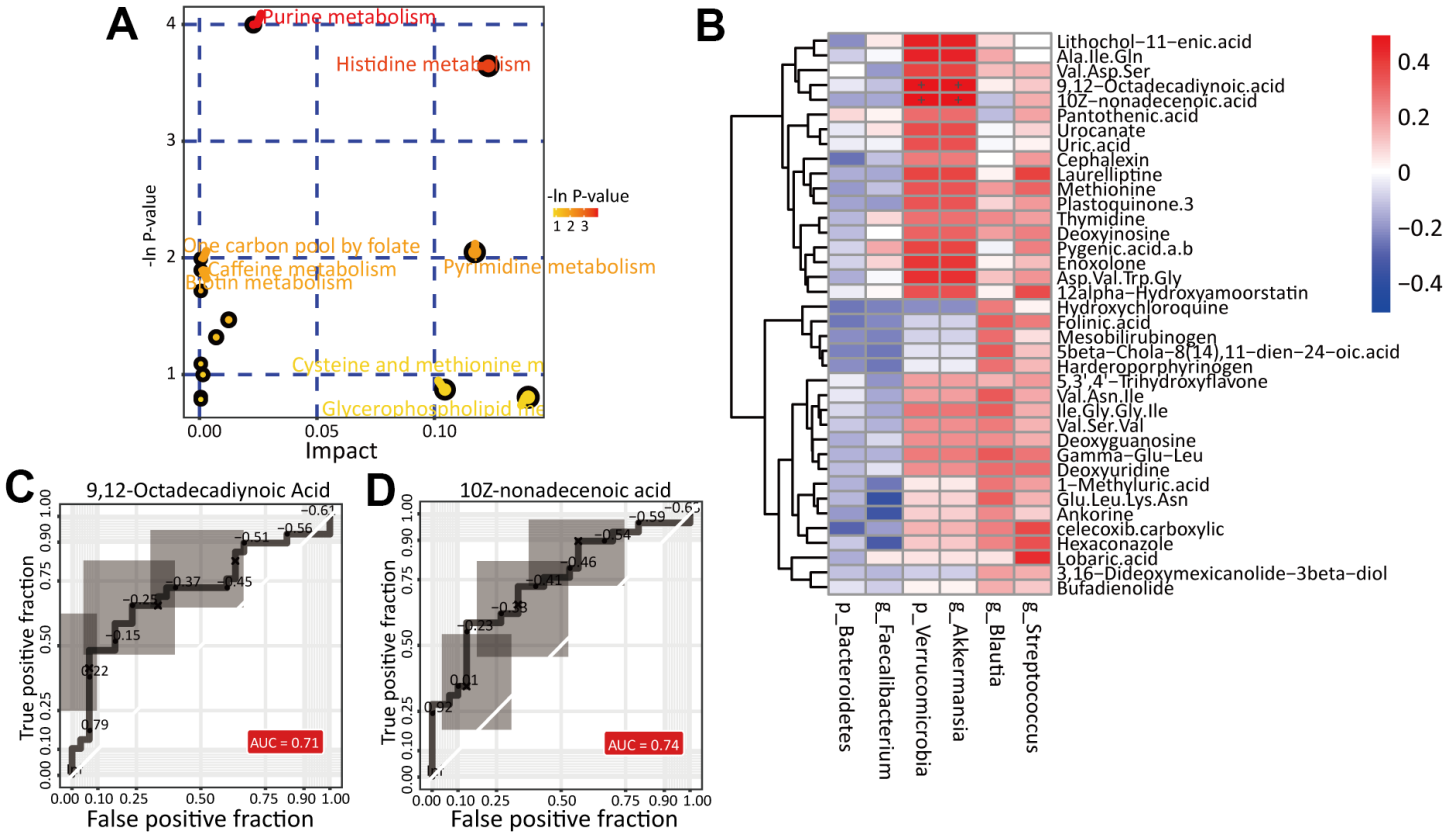
**Identification of potential biomarkers.** (**A**) The pathway analysis of differential metabolites. (**B**) Correlation analysis of differential gut microbiota and differential metabolites between HC and RA group. The ROC curve of biomarker analysis for 9,12-Octadecadiynoic Acid (**C**) and 10Z-nonadecenoic acid (**D**).

Then, we analyzed the pathways enriched in the differentially abundant metabolites mentioned above with the MetaboAnalyst website (https://www.metaboanalyst.ca/), the results of which are shown in Figure 6A. We found two pathways significantly enriched in the metabolites: the purine metabolism (p=0.01 and impact value=0.02) and histidine metabolism (p=0.02 and impact value=0.12) pathways. The metabolites xanthine, deoxyinosine, deoxyguanosine and uric acid were enriched in the purine metabolism pathway, while the metabolites urocanate and 4-imidazolaetic acid were enriched in the histidine metabolism pathway. These results suggest that alteration of the fecal microbiota leads to metabolic pathway alterations, especially in nucleic acid metabolism and amino acid metabolism.

### Correlation of the gut microbiota and fecal metabolites

According to the criteria “VIP>1.5” and “Fold Change (RA/HC)>2”, we obtained 38 metabolites whose abundance significantly differed between the HC and RA groups. Then, we performed Spearman’s correlation analysis and, based on Spearman’s correlation coefficients, found that in our study, p_Verrucomicrobia and g_Akkermansia were strongly positively correlated with 9,12-octadecadiynoic acid (r=0.54, corrected p value=0.01) and 10Z-nonadecenoic acid (r=0.54, corrected p value=0.03) (Figure 6B). Notably, both of the differentially abundant metabolites are long-chain fatty acids, which indicates that alteration of the gut microbiota may lead to long-chain fatty acid metabolic disorders, affecting the occurrence and development of RA.

Then, via receiver operating characteristic (ROC) curve analysis, we identified potential biomarkers and found that 9,12-octadecadiynoic acid (area under the curve (AUC)=0.718, p=0.0043) and 10Z-nonadecenoic acid (AUC=0.739, p=0.0064) were significantly higher in the RA group than in the HC group (Figure 6C, 6D).

## DISCUSSION

Rheumatoid arthritis is an autoimmune disease, and genetic and environmental factors play an important role in its occurrence and development. Numerous studies [17, 18] have shown that changes in the composition and function of the gut microbiota are closely related to RA; however, the influence of its components and their metabolism on RA remains unclear. In this study, using 16S rRNA gene sequencing and UHPLC-MS-based metabolomics, we assessed 30 healthy subjects and 29 RA patients to determine the effect of the gut microbiota and its metabolites on the occurrence and development of RA. The results of taxonomic analysis showed that the gut microbiota of the healthy control group and RA group significantly differed at the phylum and genus levels. We also found that the metabolites in the HC and RA groups were significantly different.

We found that α-diversity analysis showed no significant differences between the healthy control (HC) and RA groups, and the composition of the gut microbiota in the RA patients was similar to that in healthy subjects, in accordance with a previous study [19]. However, β-diversity analysis showed that the healthy subjects and the RA patients were clearly separated, suggesting that the gut microbiota of the two groups was significantly different. At the phylum level, Firmicutes, Bacteroidetes, Proteobacteria and Actinobacteria were the main components in the gut microbiota of the healthy subjects and the RA patients [20]. We found that alterations in the gut microbiota were mainly in abundance but not in composition. At the genus level, we focused on the high content of the gut microbiota and were also interested in more significantly different genera. The main cause of intestinal flora imbalance is a decrease in probiotics (such as Bifidobacterium) and an increase in pernicious bacteria. Previous studies have shown that probiotics can simultaneously significantly alleviate the progression of RA and decrease the levels of inflammatory factors [21]. In our study, Bifidobacterium was decreased in the RA group, which may have accelerated the development of RA. However, studies on Chinese patients showed that Lactobacillus was increased in cases of very active RA [22, 23]. Lactobacillus was significantly more abundant in the HC group than in the RA group. This finding suggests that a probiotic imbalance is related to the active and inactive stages of RA, but the exact functions of probiotics during the occurrence and development of rheumatoid arthritis need further investigation. A previous report showed that Prevotella and Bacteroides had an inverse relationship in terms of their abundance in RA, and Prevotella was increased, while Bacteroides was reduced in RA cases [11]. In this study, both Prevotella and Bacteroides were decreased in the RA patients, and the trend in Bacteroides abundance was in accordance with the results of a previous study [10]. In other research, Prevotella spp. were more abundant in newly diagnosed RA patients than in healthy subjects but less abundant in confirmed RA cases [11, 24]. In addition, previous studies showed that the phylum Bacteroidetes was enriched in early RA patients [25], and changes in the gut microbiota are thought to be related to the stage of RA development. Our research also found that Verrucomicrobia and Akkermansia were more abundant in the RA patients [26]. Another study confirmed that the class Bacilli and order Lactobacillales were increased in RA patients not receiving therapy [27], which was confirmed by our results.

Alterations in the fecal microbiota may lead to alterations in fecal metabolites. We next performed UHPLC-MS analyses and successfully detected over 1000 metabolites from the fecal samples of the healthy subjects and the RA patients (Supplementary Table 1); among these metabolites, 29 differentially abundant metabolites were acquired in NEG, and 45 differentially abundant metabolites were acquired in POS. OPLS-DA showed that the RA patient samples clearly differed from the samples from the healthy subjects, suggesting that the healthy subjects and the RA patients exhibit different metabolic phenotypes.

In our study, we identified two differentially abundant fatty acid metabolites as potential biomarkers. In previous studies, fatty acids were confirmed to be related to inflammation and increased in RA patients [28]. In addition, a new study found that various biological pathways related to metabolism, including fatty acid biosynthesis and glycosaminoglycan degradation, were enriched when groups of RA patients and healthy controls were compared [10]. Some long-chain fatty acids (such as ω-3 fatty acids) have been demonstrated to play a protective role in RA and improve clinical symptoms in RA patients [29]. Besides, short-chain fatty acids are one of microbial metabolites, which have been reported to be key regulators of the gut-joint axis in animal models [30]. In our study, long-chain fatty acids (9,12-octadecadiynoic acid and 10Z-nonadecenoic acid) were increased in the RA patients and strongly positively correlated with p_Verrucomicrobia and g_Akkermansia, indicating that these two fatty acid metabolites may affect the occurrence and development of RA. However, understanding their functions requires further study.

## MATERIALS AND METHODS

### Collection of fecal samples

Ethical approval was obtained from the Shenzhen People’s Hospital ethics committee (LL-KY-2020322). We collected fecal samples from 29 RA patients who were confirmed according to the RA diagnostic standards and 30 healthy subjects [31] in Shenzhen People’s Hospital. All subjects were informed and signed a consent form. We collected fecal samples on a clean bench in a sterile environment and then stored them in a liquid nitrogen tank for 15 minutes. Next, they were stored at -80° C in an ultralow temperature freezer for further analysis of the intestinal microbial community and metabolism.

### Gene sequencing analysis of 16S rRNA

The Mobio PowerSoil® DNA Isolation Kit (Qiagen, Hilden, Germany) was used to extract the total genomic DNA of the intestinal flora. The V3-V4 region of the 16S rRNA gene of the gut microbiota was amplified by PCR with the forward primer 5’-ACTCCTACGGAGGCAGCA-3’ and the reverse primer 5’-GGACTACHGGGGTWTCTAAT-3’. VAHTSTM DNA magnetic beads were used to purify the PCR products, after which we used the previously obtained PCR products as a DNA template to perform Solexa PCR. After purification, the DNA concentrations were determined with a NanoDrop 2000 system, and DNA fragments were recovered by 1.8% agarose gel electrophoresis (120 V, 40 min). Based on the Illumina HiSeq 2500 sequencing platform, we constructed small fragment library with the paired-end method, and sequenced it.

FLASH (version 1.2.11) was used to split the reads of every sample and obtain the raw tags. Then, the raw tags were filtered with Trimmomatic (version 0.33) software, and clean tags were obtained. UCHIME (version 8.1) software was used to remove the chimera sequences, and finally, we obtained the effective tags. Then, the tags were clustered with QIIME (version 1.8.0) software, and OTUs with a similarity higher than 97% were considered the same OTU. Then, the species of each OTU sequence was matched to a microbiological reference database (Release128, http://www.arb-silva.de) to obtain the species information of every OTU classification. Alpha diversity analysis was used to analyze the species richness (Chao1 and ACE) and diversity (Shannon and Simpson indices) of the gut microbiota with the online software Motheu (version 1.30, http://www.mothur.org/). Beta diversity analysis, including principal coordinate analysis (PCoA), was used to analyze differences in the species composition of the gut microbiota among different samples by R language. Linear discriminant analysis (LDA) effect size (LEfSe) analysis was used to identify biomarkers with significant differences between the two groups (HC and RA).

### Fecal metabolite analysis

### LC-MS analysis

LC-MS analyses were performed using an UHPLC system (1290, Agilent Technologies) coupled to Q Exactive (Orbitrap MS, Thermo) and an HSS T3 UPLC column (2.1 mm × 100 mm, 1.8 μm). Mobile phase A was 5 mmol/L ammonium acetate in water for NEG model and 0.1% formic acid for POS mode, and mobile phase B was acetonitrile. The elution gradient was as follows: 0 min, 1% acetonitrile; 1 min, 1% acetonitrile; 8 min, 99% acetonitrile; 10 min, 99% acetonitrile; 10.1 min, 1% acetonitrile; and 12 min, 1% acetonitrile. The acquisition software (Xcalibur 4.0.27, Thermo) continuously evaluates the full-scan survey MS data as it collects and triggers the acquisition of MS/MS spectra depending on preselected criteria. The ESI source conditions were as follows: sheath gas flow rate of 45 arb, aux gas flow rate of 15 arb, capillary temperature of 400° C, full MS resolution of 70,000, MS/MS resolution of 17,500, collision energy of 20/40/60 eV in the NCE model, and spray voltage of 4.0 kV for POS and -3.6 kV for NEG.

### Data analysis

Then, with ProteoWizard, the raw data were converted to the mzXML format and processed by MAPS software (version 1.0). The preprocessing results generated a data matrix that consisted of the retention time (RT), mass-to-charge ratio (m/z) values, and peak intensity from which the fecal metabolites were identified. The in-house MS2 database was used for metabolite identification. OPLS-DA was carried out with SIMCA software (V15.0.2, Sartorius Stedim Data Analytics AB, Umea, Sweden). Differentially abundant metabolites between the healthy subject (HC) group and the RA group were obtained by selection according to the variable importance in the projection (VIP) score and the p value from analysis by Student’s t-test. The metabolites were identified according to the accurate molecular weight and fragment pattern by the in-house MS2 database and by comparison to the following online databases: the Human Metabolome Database (HMDB; http://www.hmdb.ca/) and PubChem (https://pubchem.ncbi.nlm.nih.gov/).

## Supplementary Material

Supplementary Figures

Supplementary Table 1A

Supplementary Table 1B

Supplementary Table 1C

Supplementary Table 1D

Supplementary Table 1E

Supplementary Table 1F

Supplementary Table 1G
