# Supplementary material for: Analysis of gut microbiota and metabolites in patients with rheumatoid arthritis and identification of potential biomarkers
Source: Aging (Albany NY). 2021 Oct 20;13(20):23689–701. doi: 10.18632/aging.203641 (PMC8580343; doi:10.18632/aging.203641)
Supplement: Supplementary Figures [file aging-13-203641-s001.pdf]

SUPPLEMENTARY FIGURES

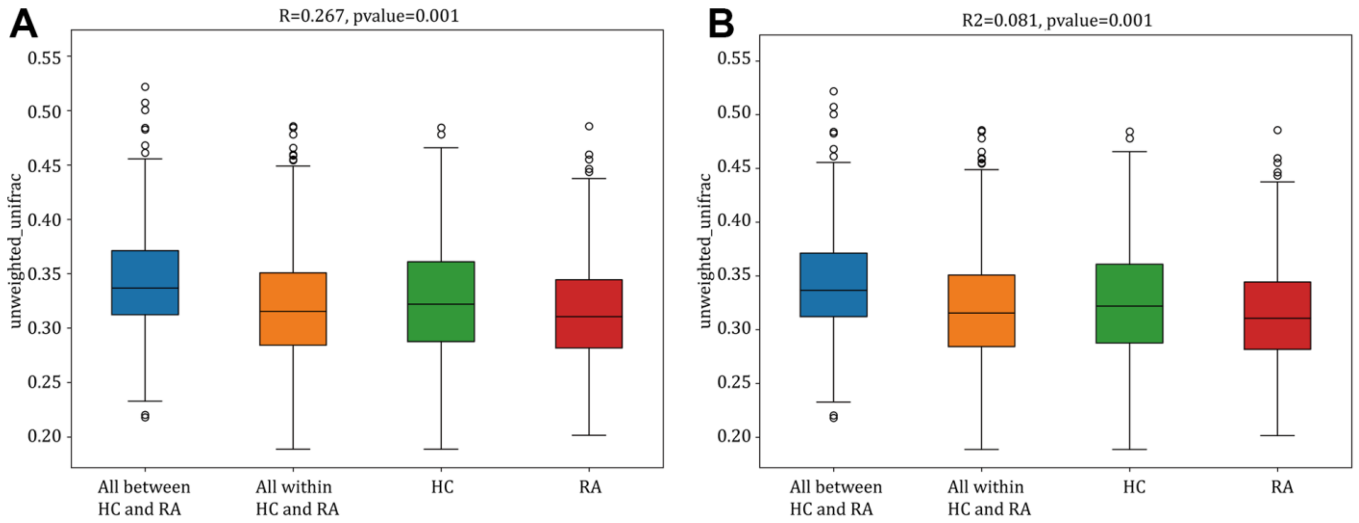

Supplementary Figure 1. β-diversity analysis showed that there was a significant difference between HC and RA group. (**A**) PERMANOVA analysis (p = 0.001), (**B**) ANOSIM analysis (p =0.001).

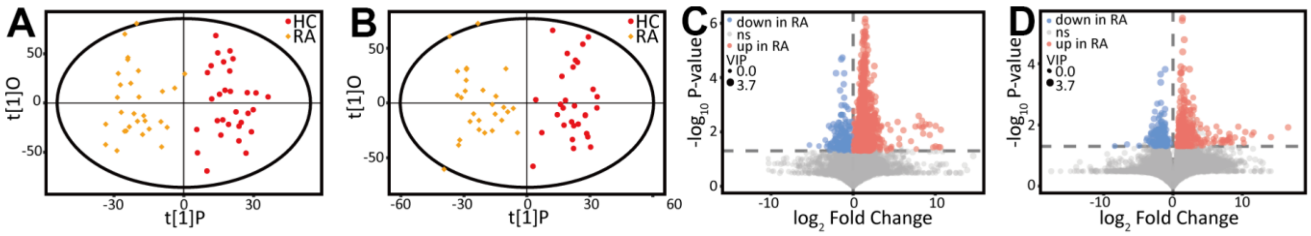

Supplementary Figure 2. OPLS-DA score plots of fecal samples. (**A**) Negative ion mode, and positive ion mode (**B**), one dot represent one sample. Volcano plot of fecal samples. (**C**) Negative ion mode and (**D**) positive ion mode. Red dot represent significantly up-regulated in RA group, blue dot represent significantly down-regulated in RA group, one dot represent one differential metabolite.
